# Association Study to Evaluate FoxO1 and FoxO3 Gene in CHD in Han Chinese

**DOI:** 10.1371/journal.pone.0086252

**Published:** 2014-01-28

**Authors:** Ying Zhao, Yanbo Yu, Xiaoli Tian, Xi Yang, Xueqi Li, Feng Jiang, Yundai Chen, Maowei Shi

**Affiliations:** 1 Department of Geriatrics, Jinan Military General Hospital, Jinan, China; 2 Department of Gastroenterology, Qilu Hospital of Shandong University, Jinan, China; 3 Department of Human Population Genetics, Institute of Molecular Medicine, Peking University, Beijing, China; 4 Department of Cardiology, the Fourth Affiliated Hospital of Harbin Medical University, Harbin, China; 5 Department of Cardiology, Chinese PLA General Hospital, Beijing, China; Sanjay Gandhi Medical Institute, India

## Abstract

**Background:**

Coronary heart disease (CHD) is one of the leading causes of mortality and morbidity in China. Genetic factors that predispose individuals to CHD are unclear. In the present study, we aimed to determine whether the variation of FoxOs, a novel genetic factor associated with longevity, was associated with CHD in Han Chinese populations.

**Methods:**

1271 CHD patients and 1287 age-and sex-matched controls from Beijing and Harbin were included. We selected four tagging single nucleotide polymorphisms (SNPs) of FoxO1 (rs2755209, rs2721072, rs4325427 and rs17592371) and two tagging SNPs of FoxO3 (rs768023 and rs1268165). And the genotypes of these SNPs were determined in both CHD patients and non-CHD controls.

**Results:**

For population from Beijing, four SNPs of FoxO1 and two SNPs of FoxO3 were found not to be associated with CHD (p>0.05). And this was validated in the other population from Harbin (p>0.05). After combining the two geographically isolated case-control populations, the results showed that the six SNPs did not necessarily predispose to CHD in Han Chinese(p>0.05). In stratified analysis according to gender, the history of smoking, hypertension, diabetes mellitus, hyperlipidemia and the metabolic syndrome, we further explored that neither the variants of FoxO1 nor the variants of FoxO3 might be associated with CHD (p>0.05).

**Conclusion:**

The variants of FoxO1 and FoxO3 may not increase the prevalence of CHD in Han Chinese population.

## Introduction

Coronary heart disease (CHD) is the most common cause of death in China and accounts for approximately one third of all deaths around the world. Multiple risk factors are involved in the cause of CHD, including modifiable factors (life styles, smoking, hypertension, hyperlipidemia, diabetes), and fixed factors (aging, gender, genetic predisposition) [1]. Over decades, the mechanisms of oxidative stress and inflammation in association with endothelial dysfunction and vascular smooth muscle proliferation have been investigated by numerous studies, with reactive oxygen species (ROS) being the common mechanism by which different CHD risk factors trigger atherosclerosis [2], [3]. All these events can be regulated by transcription factors which control the expression of genes associated with the progression of CHD. Therefore, identification of possible transcription factors related with the CHD process may help in efforts to decrease the risk of the disease.

Forkhead transcription factors of the O class (FoxOs), primarily identified as downstream targets of insulin/IGF-1 signaling pathway, consist of four members, FoxO1, FoxO3, FoxO4 and FoxO6 [4]. These transcription factors have been identified as important regulators involved in cellular differentiation, apoptosis, oxidative stress, glucose metabolism and other cellular functions [5], [6]. Furthermore, FoxOs are increasingly considered as potential clinical targets for multiple disorders since they modulate the expression of genes associated with metabolic disease [7], [8], cardiovascular injury [9], neurodegeneration [10], tumorigenesis [11] and cell longevity [12].

Exploring the genetic contribution to the pathogenesis of CHD has been considered important step for the medical intervention of the disease. With regard to the research on the genetics of FoxOs, it was firstly reported in human soft tissue tumors and leukemias [13], [14]. Recent genome-wide association study in the Framingham population indicated that FoxO1 was strongly associated with the age of death [15]. In addition, several association studies on human longevity highlighted the role of FoxO3 in Japanese, German and Southern Italian [16]–[18]. It is probable that FoxO1 and FoxO3 affect longevity through multiple mechanisms, such as insulin resistance, stress responses or proneness to disease. In the cardiovascular system, FoxOs regulate multiple aspects of cellular function in vascular tissues. And FoxO1 and/or FoxO3 are/is considered as apoptosis-regulating gene for the onset of diabetic cardiomyopathy [19], cardiac hypertrophy [20] and ischemic heart disease [21]. It has been reported that the upregulation of FoxO1 and FoxO3 appears to disrupt cardiac hypertrophy [22], [23]. In a balloon carotid arterial injury rat model, gene transfer of FoxO3 can inhibit vascular smooth muscle cell proliferation and neointimal hyperplasia [24]. The expression of FoxO1 can be stimulated by α1-adrenergic agonists and ultimately lead to apoptotic endothelial cell injury [25]. Considering the possible role of FoxO1 and FoxO3 in the maintenance of vascular homeostasis, in the present study we aimed to investigate the intrinsic association of FoxO1 and FoxO3 with CHD phenotype in Han Chinese. We selected four tagging SNPs of FoxO1 (rs2755209, rs2721072, rs4325427 and rs17592371) and two tagging SNPs of FoxO3 (rs768023, rs1268165). The frequencies of FoxO1 and FoxO3 were testified in Chinese CHD patients from two different regions.

## Materials and Methods

### 1. Subjects

The cases of this study were all hospitalized patients recruited from two medical centers in Beijing and Harbin. All the CHD patients were diagnosed by one of the following standards: (1) based on World Health Organization criteria in terms of elevations of cardiac enzymes, electrocardiography and clinical symptoms; (2) angiographic evidence of more than 50% stenosis in one or more major coronary arteries; (3) previous history of PCI (percutaneous coronary intervention) or CABG (coronary artery bypass graft). The control subjects matched with the patients for age and sex. And they were recruited from the two medical centers meeting the following criteria: (1) with no family history of CHD in first-degree relatives before the age of 60 in male and the age of 55 in female; (2) no clinical symptoms for CHD; (3) resting electrocardiography(ECG) showed normal results. The participants with no less than 2 risk factors of CHD (age>45 for man and >55 for woman, hypertension, diabetes, overweight, hyperlipidemia) were subjected to treadmill stress test and selected as controls if their ECG were normal without clinical symptoms. Those with history of cardiomyopathy, valvular disease, peripheral vascular disease, stroke, severe hepatic and kidney deficiency were ruled out.

Smokers were defined as individuals who were smoking or who had stopped<1 years before they were enrolled in this study based on self-reports.

Hypertension was diagnosed by the following criteria: (1) at present receiving antihypertensive therapy; (2)blood pressure≥140/90 mmHg confirmed at least three times of different days.

Diabetes mellitus was defined by: (1) taking hypoglycemic agents; (2) fasting serum glucose≥7.0 mmol/L, 2 h postprandial glucose level≥11.1 mmol/L in two measurements; (3) oral glucose tolerance test confirmed.

Hyperlipidemia was defined by: (1) taking lipid-reducing treatment (2) total cholesterol ≥200 mg/dL or low density lipoprotein cholesterol≥130 mg/dL.

Body mass index (BMI) was calculated by the formula: body weight (Kg)/height^2^ (m^2^).

The metabolic syndrome (MetS) was diagnosed by three or more of the following criteria: elevated WC (waist circumference) (for Chinese, the cut points for WC were ≥85 cm in men and ≥80 cm in women); TG≥1.7 mmol/l; HDL-C<1.0 mmol/l in men and <1.3 mmol/l in women; BP≥130/85 mmHg or on antihypertensive drug treatment in a patient with a history of hypertension; or FPG≥5.6 mmol/l.

Written informed consent was obtained from all individuals, and the study protocol was approved by the Ethics Committee of the PLA General Hospital and the Ethics Committee of Harbin Medical University. The study conformed to the principles outlined in the Declaration of Helsinki.

### 2. genotyping

Venous blood was collected by standard vein puncture in fasting condition and without intake of any medication either at the time of acute coronary event onset or at most for 2 weeks. Human genomic DNA was extracted from EDTA-anticoagulated blood using the proteinase K methods described previously [26]. Based on the hapmap(CHB+JPT), the four tagging SNPs of FoxO1 (rs2755209, rs2721072, rs4325427 and rs17592371) and the two tagging SNPs of FoxO3 (rs768023, rs1268165) were selected. The selections of the SNPs were based on the following criteria: (1) tag SNPs based on r^2^≥0.8; (2) functional position; (3)minor allele frequency>10%. DNA fragments of 120–180 bp containing SNPs were amplified by PCR from 10 ng of genomic DNA from each participant, with the primers listed (Table 1). The amplified DNA fragments were purified and used for genotyping by direct-sequencing with a BigDye v3.1 kit and running on ABI 3130XL.

**Table 1:** The pairs of PCR primers for amplifications of SNPs for FoxO1 and FoxO3.

SNP	Gene	Position	primer
rs2755209	FoxO1	intron	forward: 5′-CTCACCTCGAGACACGCTGT-3′
			reverse: 5′-GGATCTTAGGGGAATGCAAT-3′
rs2721072	FoxO1	intron	forward: 5′-CCCACATGAGAACCCTGTAT-3′
			reverse: 5′-CTGATAGTGCATAGAGCCCAT-3′
rs4325427	FoxO1	intron	forward: 5′-CATACAGTCAGCGAATGTCCT-3′
			reverse: 5′-GACAGGCATGAGAGATACCT-3′
rs17592371	FoxO1	3′UTR	forward: 5′-CCAGTGTAGTGACCCAAGTAT-3′
			reverse: 5′-GATAGTTTTCAGCGCTGGGT-3′
rs768023	FoxO3	promoter	forward: 5′-GGAACCAGAGAGTCAGAGCT-3′
			reverse: 5′-GGTCTGGCATTGACTGATTGT-3′
rs1268165	FoxO3	downstream	forward: 5′-GAGAGCTGAGTTGGTCACCT-3′
			reverse: 5′-GCATGGTAATTCTAGAACTGTT-3′

### 3. Statistical analysis

Continuous data was expressed as mean±standard deviation. The differences in general characteristics between case and control were compared by student t test for quantitative variables and chi-square test for categorical variables. Chi-square test was also used to test for deviation of genotype distribution from Hardy-Weinberg equilibrium. All statistical analysis was performed with SPSS 17.0. HaploView 4.2 was used to analyze linkage disequilibrium (LD), and LD was demonstrated by r^2^ value. r^2^≥0.8 indicated the SNPs were in a same natural haplotype block. A *P*-value<0.05 was considered statistically significant.

## Results

### 1. Characteristics of population

Baseline characteristics of all subjects were presented in Table 2. CHD patients and non-CHD controls were collected from two medical centers in north-eastern and northern China. All participants were Han Chinese. Population 1 was from Beijing consisting of 808 cases and 829 age- and sex-matched non-CHD controls. Population 2 was from Harbin comprising 463 cases and 458 age- and sex-matched non-CHD controls. Significant differences were found between cases and controls in Population 1 and 2 in terms of BMI, smoking, hypertension, diabetes mellitus, hyperlipidemia and MetS.

**Table 2:** Characteristics of populations.

	Population 1			Population 2		
	case (n = 808)	control (n = 829)	P value	case (n = 463)	control (n = 458)	P value
age (year)	60.36±10.22	61.12±12.01	0.166	54.06±8.76	53.27±9.06	0.175
male	634 (78.5%)	647 (78.0%)	0.837	335 (72.4%)	332 (72.5%)	0.963
BMI (kg/m^2^)	25.70±3.28	24.97±3.08	<0.001	25.56±3.26	24.20±2.89	<0.001
smoking	367 (45.4%)	111 (13.4%)	<0.001	269 (58.1%)	232 (50.7%)	<0.001
Hypertension	528 (65.3%)	311 (37.5%)	<0.001	294 (63.5%)	118 (25.8%)	<0.001
diabetes mellitus	225 (27.8%)	104 (12.5%)	<0.001	125 (27.0%)	30 (6.6%)	<0.001
hyperlipidemia	439 (54.3%)	521 (62.8%)	<0.001	314 (67.8%)	181 (39.5%)	<0.001
Metabolic syndrome	272 (33.7%)	163 (19.7%)	<0.001	142 (30.7%)	50 (10.9%)	<0.001

The data were presented as mean±SEM (standard error of the mean) for age and BMI as well as No.(percentage) for other factors. P values for age and BMI were calculated from t-test comparing case and control groups within population. P values for gender, smoking, hypertension, diabetes mellitus, hyperlipidemia, metabolic syndrome were calculated from Chi-square test within population. BMI: body mass index.

### 2. Genotype distribution and allelic frequencies

In both two groups from different regions, there was no significant deviation for four tagging SNPs of FoxO1 and two tagging SNPs of FoxO3 by the Hardy-Weinberg equilibrium test. To test the genotype association between FoxO1/FoxO3 and CHD, we performed Chi-square test (table 3). For population from Beijing, six SNPs of FoxO1 and FoxO3 were found not to be associated with CHD (p>0.05). And this was validated in the other population from Harbin (p>0.05). After combining the two geographically isolated case-control populations, the results showed that the six SNPs did not necessarily predispose to CHD in Han Chinese(p>0.05). We further conducted stratification analysis according to gender, smoking, medical history of hypertension, diabetes mellitus, hyperlipidemia and MetS, and no obvious association between genotype distribution and CHD was observed in CHD patients and non-CHD controls (Table S1, Table S2, Table S3, Table S4, Table S5 and Table S6).

**Table 3:** Frequency of FoxO1 and FoxO3 polymorphism in CHD from two different populations.

		Subjects from Population 1			Subjects from Population 2			Combined subjects		
SNP	genotype	CHD	Non-CHD	P	CHD	Non-CHD	P	CHD	Non-CHD	P
rs2755209	CC	403	423	0.269	221	223	0.836	624	646	0.234
	CA	319	301		209	199		528	500	
	AA	86	105		33	36		119	141	
Allelic A frequency		0.304	0.308		0.297	0.296		0.301	0.303	
rs2721072	AA	360	399	0.285	219	207	0.245	579	606	0.263
	AG	342	336		179	199		521	535	
	GG	106	94		65	52		171	146	
Allelic G frequency		0.343	0.316		0.334	0.331		0.339	0.321	
rs4325427	TT	378	392	0.393	226	220	0.759	604	612	0.322
	TC	326	348		185	192		511	540	
	CC	104	89		52	46		156	135	
Allelic C frequency		0.330	0.317		0.312	0.310		0.324	0.325	
rs17592371	CC	380	387	0.986	218	202	0.651	598	589	0.794
	CT	297	308		181	191		478	499	
	TT	131	134		64	65		195	199	
Allelic T frequency		0.346	0.347		0.334	0.350		0.341	0.348	
rs768023	AA	579	565	0.087	307	301	0.937	886	866	0.243
	AG	176	218		136	135		312	353	
	GG	53	46		20	22		73	68	
Allelic G frequency		0.175	0.187		0.190	0.195		0.180	0.189	
rs1268165	TT	484	515	0.424	301	302	0.526	785	817	0.247
	TC	296	280		142	130		438	410	
	CC	28	34		20	26		48	60	
Allelic C frequency		0.218	0.210		0.197	0.199		0.210	0.206	

Calculations are performed with comparison of three different genotypes. Values are the number of subjects. No significant difference (chi-square test) was found in the frequency of either polymorphism between CHD cases and non-CHD controls.

## Discussion

In the present study, we identified four tagging SNPs of FoxO1 and two tagging SNPs of FoxO3 with CHD in two geographically isolated Han Chinese populations. And our data showed that these six investigated SNPs of FoxO1/FoxO3 might not be distributed differently between CHD patients and non-CHD controls in population from Beijing and Harbin. Stratification analysis was carried out to understand the interaction between genetic and other risk factors, and the addition of other risk factors seemed not influencing the susceptibility for CHD. These results indicated for the first time that the association of FoxO1/FoxO3 with the risk of CHD was not statistically significant in Han Chinese.

How the genetic determinants contribute to CHD has provoked great interest in recent years. These population-based genome-wide association studies (GWAS) were trying to identify specific genotypes and alleles responsible for CHD. As is known, FoxOs, acting as important heredity factors in aging, are important regulators involved in the process of oxidative stress, immune surveillance, vascular tone and cardiovascular development. Besides, FoxOs can modulate the metabolic environment by regulating the expressions of specific enzyme and energy-dependant proteins [27]. Several lines of evidence demonstrated that FoxO1 and FoxO3 were expressed in murine heart and coronary arteries [28]–[30]. Altered FoxO1 function in vascular endothelial cells was reported to be responsible for the observed worsening of lesions [31]. FoxO3 can regulate the expression of certain factors in cardiac fibroblasts, such as peroxiredoxin III-a cardioprotectant [32]. Furthermore, FoxO3 is also expressed in endothelial cells, and it can modulate endothelial cell migration and sprouting during vascular development. However, in our genotyping research, neither FoxO1 nor FoxO3 was testified to be associated with CHD. It may be explained with considerations as follows.

Atherosclerosis in CHD is often confused with vascular aging. Aged vessels show a number of characteristic pathological processes (reduced medial VSMC number, increased collagen deposition, fracture of the elastin lamellae, etc), many of which are also seen in atherosclerosis. However, by the standards of pathology, arteriosclerosis is divided into three types: atherosclerosis of large and medium-sized arteries, monckeberg medical calcific sclerosis of medium-sized arteries, and arteriolosclerosis. Thus, vascular aging is not included among the three types of arteriosclerosis. Vascular aging is a process characterized by various alternations in a physical environment of cells in vessels [33]. Oxygen free radicals and mitochondrial DNA mutations have been closely associated with vascular aging. It is of interest to note that the incidence of atherosclerosis increases with advancing age and aging is a strong risk factor for atherosclerosis [34]. However, large numbers of population-based prospective studies have shown that atherosclerosis is a complex syndrome closely associated with uncontrollable and controllable factors, such as age, gender, smoking, obesity and diabetes mellitus. Although several studies provided promising findings in the association of FoxOs gene with the aging process, due to the various risk factors and the complexity of CHD, the role of FoxOs on the pathogenesis of CHD has not been identified in our study

Oxidative stress plays an important role in the process of atherosclerosis. The dysregulated oxidant and antioxidant balance brings about the alterations in redox status, and subsequently leads to VSMC proliferation, endothelial dysfunction, inflammatory response and lipid peroxidation [35], [36]. All these detrimental events result in vessel wall thickness and vascular remodeling which induce a susceptibility to CHD. However, mounting evidence supports that chronic inflammation plays a central role in the pathogenesis of CHD, which is recognized to occur from the earliest stages of atheroma formation through to plaque rupture and thrombosis [37], [38]. Cytokines secreted by inflammatory cells (T cells, mast cells and monocytes) could contribute to the initiation, development and rupture of atherosclerotic plaque [39]–[41]. Therefore, the relative balance of these inflammatory processes will predict the development of CHD. Although CHD is age-associated vascular disease, the contribution of inflammatory cells and mediators in the pathogenesis of CHD should also be emphasized.

One of the major biochemical pathways playing a role in the inflammatory process is the NF-κB signaling pathway. Using apoE^−/−^ mice, genetic suppression of NF-κB signaling led to a reduction in the size of atherosclerotic lesions [42]. Interestingly, FoxOs have been reported to suppress NF-κB signaling, providing support for the possible vasculoprotective effects of FoxOs [43]. However, we did not observe any association between FoxO1/FoxO3 variants and CHD. It should be noteworthy that atherosclerosis results from a combination of endothelial, hematopoietic, T-cell and macrophage dysfunction [44], [45]. Thereby, the modulation is very complex. Besides FoxOs, a wide range of extracellular immune stimuli, such as IL-1, IL-6, TNF-α, T-cell receptor and B-cell receptor (TCR and BCR), can mediate the regulation of NF-κB activity [45], [46]. Moreover, it has been reported that genetic polymorphisms/variations in expression of FoxO genes appear to correlate with human autoimmune disease susceptibility and or activity (such as lupus and rheumatoid arthritis) [47], [48]. Of note, the inflammation in CHD, with a special name of “metabolic inflammation”, has unique features compared to autoimmune diseases. The metabolic inflammation is mainly associated with overnutrition-induced metabolic derangements [49], . May these explain the negative indications for our association study between FoxOs and CHD.

FoxOs have been reported to play a major role in the transcriptional regulation of many proteins which are directly involved in metabolism [51]–[53]. Thereby, we also analyzed whether any of the selected SNPs in FoxO1/FoxO3 is associated with gender, smoking, medical history of hypertension, diabetes mellitus, hyperlipidemia and MetS in our study population. But we did not observe any significant association. However, to rule out any association of FoxO1/FoxO3 with CHD, additional studies are required in different populations with different allele frequencies.

In conclusion, we demonstrate that neither FoxO1 nor FoxO3 is associated with CHD in two geographically isolated Han Chinese populations. However, the number of participants in this study is relatively small, and the findings need to be cautious. A multi-center research needs to be carried out to further assess the association of FoxOs with CHD in more ethnic groups and in larger populations.

## Supporting Information

Table S1 Frequencies of FoxO1 and FoxO3 polymorphisms in two populations according to different genders.
(DOC)

Table S2 Frequencies of FoxO1 and FoxO3 polymorphisms in two populations according to smoking or not.
(DOC)

Table S3 Frequencies of FoxO1 and FoxO3 polymorphisms in two populations according to hypertension or not.
(DOC)

Table S4 Frequencies of FoxO1 and FoxO3 polymorphisms in two populations according to DM or not.
(DOC)

Table S5 Frequencies of FoxO1 and FoxO3 polymorphisms in two populations according to hyperlipidemia or not.
(DOC)

Table S6 Frequencies of FoxO1 and FoxO3 polymorphisms in two populations according to MetS or not.
(DOC)
