# Supplementary material for: Association Study to Evaluate FoxO1 and FoxO3 Gene in CHD in Han Chinese
Source: PLoS One. 2014 Jan 28;9(1):e86252. doi: 10.1371/journal.pone.0086252 (PMC3904908; doi:10.1371/journal.pone.0086252)
Supplement: Table S6 — Frequencies of FoxO1 and FoxO3 polymorphisms in two populations according to MetS or not. (DOC) [file pone.0086252.s006.doc]

Table S6. Frequencies of FoxO1 and FoxO3 polymorphisms in two populations according to MetS or not.

| SNP | genotype | Population 1 | | | | | | Population 2 | | | | | |
| --- | --- | --- | --- | --- | --- | --- | --- | --- | --- | --- | --- | --- | --- |
|  |  | MetS | | | Non-MetS | | | MetS | | | Non-MetS | | |
|  |  | CHD | Non-CHD | *P* | CHD | Non-CHD | *P* | CHD | Non-CHD | *P* | CHD | Non-CHD | *P* |
| rs2755209 | CC | 147 | 91 | 0.390 | 256 | 332 | 0.063 | 81 | 27 | 0.583 | 140 | 196 | 0.492 |
|  | CA | 96 | 61 |  | 223 | 240 |  | 55 | 19 |  | 154 | 180 |  |
|  | AA | 29 | 11 |  | 57 | 94 |  | 6 | 4 |  | 27 | 32 |  |
| rs2721072 | AA | 130 | 81 | 0.921 | 230 | 318 | 0.205 | 79 | 25 | 0.414 | 140 | 182 | 0.065 |
|  | AG | 108 | 63 |  | 234 | 273 |  | 52 | 18 |  | 127 | 181 |  |
|  | GG | 34 | 19 |  | 72 | 75 |  | 11 | 7 |  | 54 | 45 |  |
| rs4325427 | TT | 129 | 79 | 0.953 | 249 | 313 | 0.437 | 81 | 29 | 0.790 | 145 | 191 | 0.595 |
|  | TC | 107 | 64 |  | 219 | 284 |  | 48 | 15 |  | 137 | 177 |  |
|  | CC | 36 | 20 |  | 68 | 69 |  | 13 | 6 |  | 39 | 40 |  |
| rs17592371 | CC | 132 | 93 | 0.214 | 248 | 294 | 0.612 | 81 | 28 | 0.533 | 137 | 174 | 0.640 |
|  | CT | 109 | 56 |  | 188 | 252 |  | 51 | 16 |  | 130 | 175 |  |
|  | TT | 31 | 14 |  | 100 | 120 |  | 10 | 6 |  | 54 | 59 |  |
| rs768023 | AA | 236 | 132 | 0.268 | 343 | 433 | 0.211 | 73 | 24 | 0.573 | 234 | 277 | 0.257 |
|  | AG | 31 | 27 |  | 145 | 191 |  | 65 | 23 |  | 71 | 112 |  |
|  | GG | 5 | 4 |  | 48 | 42 |  | 4 | 3 |  | 16 | 19 |  |
| rs1268165 | TT | 176 | 107 | 0.963 | 308 | 408 | 0.210 | 69 | 24 | 0.719 | 232 | 278 | 0.434 |
|  | TC | 85 | 49 |  | 211 | 231 |  | 66 | 22 |  | 76 | 108 |  |
|  | CC | 11 | 7 |  | 17 | 27 |  | 7 | 4 |  | 13 | 22 |  |

Calculations were performed with comparison of three different genotypes. Values are the number of subjects. After stratification analysis according to metabolic syndrome, no significant association was found between genotype distributions and CHD in CHD patients and non-CHD controls.
